# The Structural Stability of Enzymatic Proteins in the Gas Phase: A Comparison of Semiempirical Hamiltonians and the GFN-FF

**DOI:** 10.3390/molecules30102131

**Published:** 2025-05-12

**Authors:** Jarosław J. Panek

**Affiliations:** Faculty of Chemistry, University of Wrocław, ul. F. Joliot-Curie 14, 50-383 Wrocław, Poland; jaroslaw.panek@uwr.edu.pl

**Keywords:** protein structure, gas phase, semiempirical methods, force fields, trypsin, sterol demethylase

## Abstract

The study of the gas-phase behavior of proteins has recently gained momentum due to numerous prospective applications in, e.g., the construction of molecular sensors or nano-machines. The study of proteins outside their standard water environment, necessary to arrive at their successful applied use, is, however, limited by the loss of the structure and function of the macromolecules in the gas phase. We selected two enzymatic proteins with great potential for applied use, the digestive enzyme trypsin and the cytochrome sterol demethylase, for which to develop gas-phase structural models. The employed levels of theory were semiempirical, density functional tight binding, and polarizable force-field techniques. The convergence of the self-consistent field equations was very slow and in most cases led to oscillatory behavior, encouraging careful tuning of the convergence parameters. The structural optimization and molecular dynamics simulations indicated the parts of the proteins most prone to structural distortion under gas-phase conditions with unscreened electrostatics. This problem was more pronounced for cationic trypsin, for which the stability of the simulation was lower. The fate of the hydrogen bonding network of the catalytic triad in the gas phase was also investigated.

## 1. Introduction

Proteins, complex systems composed of amino acid chains and frequently cofactors of diverse types (small molecules, nucleic acids, lipids, and carbohydrates), are associated with an aqueous environment. Some proteins are anchored into lipid bilayers to serve as channels and receptors. It is recognized that the inclusion of solvation effects is crucial for the proper prediction of folding and de novo protein design [1]. However, advancements in the experimental techniques as early as at the end of the 20th century allowed for the desolvation of macromolecules and provided insights into the behavior of proteins in the gas phase [2]. Surprisingly, many of the structural aspects of protein complexes have been preserved in the high-vacuum conditions of mass spectrometers [3]. This phenomenon can partly be attributed to the partial conservation of hydrophobic effects, which has been noted in the solution–gas-phase transfer for cyclodextrin and adamantyl complexes with the use of soft cold-spray ionization [4]. This has given rise to elaborations on the future applied role of gas-phase protein assemblies, including structural genomics and proteomics on the basis of protein complexes from cell isolates [5]. A decade ago, comprehensive reviews devoted to the fate of proteins in the gas phase began to appear. Among them, one classical work by Meyer et al. described the desolvation process itself, the experimental methods of structural investigation, and theoretical models relevant to the vacuum conditions [6]. In just a decade, gas-phase sensors containing proteins, peptides, DNA, and molecularly imprinted polymers have been proposed, with the aim of detecting volatile organic compounds and developing an artificial sense of smell [7,8]. The use of gas-phase protein structures in practical applications is therefore in its starting stage. This calls for the urgent development and validation of both experimental and theoretical approaches to the relevant structural and physicochemical investigations.

### 1.1. Experimental Approaches to Gas-Phase Protein Structures

Currently, there are experimental methods allowing for the investigation of neutral proteins in the gas phase (molecular beams). These methods are based on laser UV spectroscopy, including resonant two-photon ionization and diverse IR techniques [9]. However, the advent of gas-phase protein investigations and the majority of their experimental developments have been based on electron spray ionization combined with mass spectrometry (ESI-MS), requiring the particles to be charged. Elaborate techniques have been proposed for increasing the selectivity and improving the detection thresholds—for example, Fourier transform ion cyclotron resonance mass spectrometry combined with gas-phase fluorescence using rhodamine 6G as a fluorescence label [10]. Another emerging technique that both enhances the fluorescence efficiency and allows for the measurement of the interatomic distances is Förster resonant energy transfer (FRET), which has been known for decades but was only recently adapted to gas-phase conditions [11]. The use of two probes, donor and acceptor chromophores (one of which can be a transition metal [12]), makes it possible to relate the fluorescence efficiency to the donor–acceptor separation, even in the range of tens of Ångstroms. This short and non-exhaustive list of experimental techniques must be concluded with the statement that the exact nature of the protein structures in the gas phase cannot currently be resolved to such a high degree as that offered by diffractometry, cryo-electron microscopy, or solution NMR measurements, although X-ray free-electron lasers (such as the DESY European XFEL initiative) promise the possibility of capturing the X-ray gas-phase structures of proteins [6]. This fact calls for theoretical insight into the process of desolvation and the structural dynamics of biomacromolecules.

### 1.2. Theoretical Investigations of Gas-Phase Protein Structures

Among the numerous methods of computational chemistry, the following have frequently been used to support the experimental determination of gas-phase peptide and protein structures [9]:

- Classical force fields (AMBER, CHARMM, GROMOS, etc.) utilized in conformational space exploration via Monte Carlo or molecular dynamics methods;
- Semiempirical methods for assessing the conformational landscape of smaller peptides;
- Density functional theory (DFT), especially newer functionals with dispersion corrections (such as ωB97X-D), used with triple-zeta polarized basis sets, which limits the available size of the studied peptides.

These methods are used to provide energy minima and calculate their vibrational frequencies, which can be compared to experiments. The following are the most successful methods for vibrational calculations of biomolecules and peptides [9]:

- Harmonic frequency calculations, when coupled with wavenumber scaling, can reach an accuracy better than 20 cm^−1^;
- The vibrational self-consistent field (VSCF) method of treating anharmonicity and mode coupling;
- DFT-based Born–Oppenheimer molecular dynamics with subsequent Fourier transformation of the dipole time correlation function—standard deviations of 6 cm^−1^ can be reached using this costly yet accurate approach [13,14].

DFT-based molecular dynamics approaches, with their tremendous accuracy, are, however, limited to di- or tri-peptides [14]. Access to information on larger peptides and proteins is provided using classical force fields. It has already been recognized that gas-phase protein simulations pose potential problems [15,16]:

- The lack of electrostatic screening for the solvent can lead to increased proton mobility and a large diversity of possible protonation states;
- Force fields developed for a water environment will provide charge distributions inadequate for gas-phase conditions;
- The use of periodic boundary conditions must be either switched off (which brings a heavy performance penalty, as GPU acceleration cannot be used easily) or dealt with by using the proper setting for the cutoff radius to encompass the whole system.

Even with these restrictions in mind, it is believed [16,17] that the use of standard force fields, developed for a water environment, for gas-phase simulations of biomolecules can provide surprisingly good semi-quantitative results. Classical force fields will continue to provide access to time scales not available for quantum-chemical treatments, either semiempirical or first-principle; therefore, the necessity of studying their capacity for gas-phase simulations cannot be underestimated.

### 1.3. The Rationale for the Current Study

The main objective of the current study is an attempt to test a computational protocol complementing the well-established classical force-field approach. Since the onset of computational studies of proteins in gas-phase conditions, it has become evident that the force-field parameterizations used to reproduce the solution behavior of macromolecular systems, could also be used, with care, under gas-phase conditions [6,16]. It might be necessary to use proper scaling factors for electrostatics [17] or resort to mobile proton formulations (i.e., adjustable tautomeric states) [15,18]. Unfortunately, desolvation processes, e.g., those in ESI droplets, proceed on the μs–ms time scale, which is a serious computational challenge. Models of coarse-grained protein polymers in charged water droplets have been used to investigate the desolvation processes during electrospraying [19]—inclusion of the proton transfer events was necessary for the proper outcomes. Models based on the classical force fields have also been used to investigate the dehydration and subsequent rehydration of the virus coat proteins on bacteriophage MS2 [20], proving the ability of the proteins to regain their native structure upon rehydration. However, the correlation between the solution- and gas-phase stabilities of a particular protein is not always guaranteed [21]. It is also possible to computationally observe an interesting phenomenon of structural change in a charged protein (ubiquitin) so that the charged residues are not exposed, as in water, but rather buried inside the chain [22]. Such a plethora of possible phenomena calls for robust computational techniques. The standard force fields have been used for extensive mapping of the potential energy surfaces of dipeptides, e.g., in Ref. [23], but the sheer size of the conformational space makes such a mapping impossible for larger systems. On the other hand, the already mentioned problem of movable protons might be solved through the use of quantum-mechanical approaches. Most of these are too computationally demanding, and therefore layered methods such as ONIOM [24] might prove valuable—at the price of defining the borders between the layers, i.e., the systems covered by the different levels of theory. Seemingly, a unique, universal proposal on modeling proteins in the gas phase has not yet emerged. The computational setup used within this work does not aim to be universal but rather helpful in identifying the regions of proteins which need special attention. For this purpose, full-size systems will be tackled since simplified models (e.g., short peptides) will not exhibit the necessary complexity.

This study is devoted to the use of two different kinds of semiempirical quantum-chemical methods in biomolecular simulations. The two kinds are either standard wavefunction-based semiempirical models represented by AM1 and PM3 Hamiltonians [25,26] or density functional tight binding (DFTB) in two versions, GFN1-xTB and GFN2-xTB, proposed by S. Grimme and coworkers [27,28]. Additionally, a fully automated partially polarizable force field GFN-FF [29] was selected to assess the performance of the modern force fields. We undertake quantum-chemical semiempirical simulations of biomolecular systems with 3000+ atoms to explore the potential problems associated with the charged systems in the gas phase. The two proteins selected for this study, a digestive enzyme, cationic trypsin, and a cytochrome P450 enzyme, sterol 14α-demethylase CYP51, represent enzyme classes with important biochemical roles. The potential of their application as biosensors or chemical nanoreactors will increase if reliable theoretical approaches to modeling their behavior are found—which is the second main motivation of our study.

## 2. Results and Discussion

### 2.1. A General Description of the Studied Proteins

**Figure 1:**
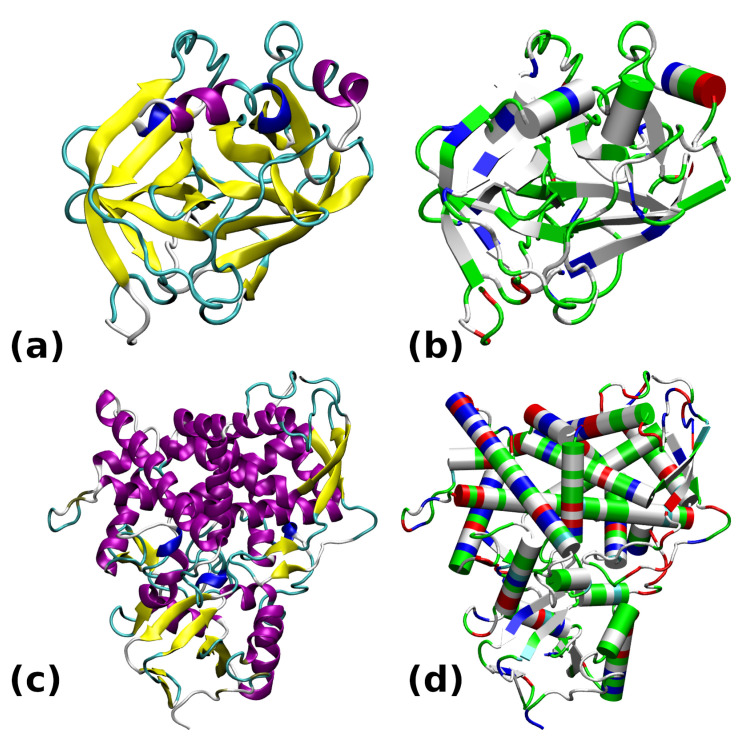
Structures of the studied proteins: (**a**) Trypsin 6B6T colored by secondary structure. (**b**) Trypsin 6B6T colored by residue types. (**c**) CYP51 5FSA colored by secondary structure. (**d**) CYP51 5FSA colored by residue types. The secondary structure color coding: α-helix—purple; 3_10_-helix—blue; β-strand—yellow; turn—cyan; random coil—white. Color coding of residue types: basic—blue; acidic—red; polar—green; apolar—white.

The two chosen proteins are deposited into the PDB structural database under the following codes: 6B6T for the cationic trypsin [30] and 5FSA for the sterol 14α-demethylase [31]. Their structures, drawn for the purpose of this study using two different coloring schemes (related either to the secondary structure or to the residue type), are presented in Figure 1.

**Figure 2:**
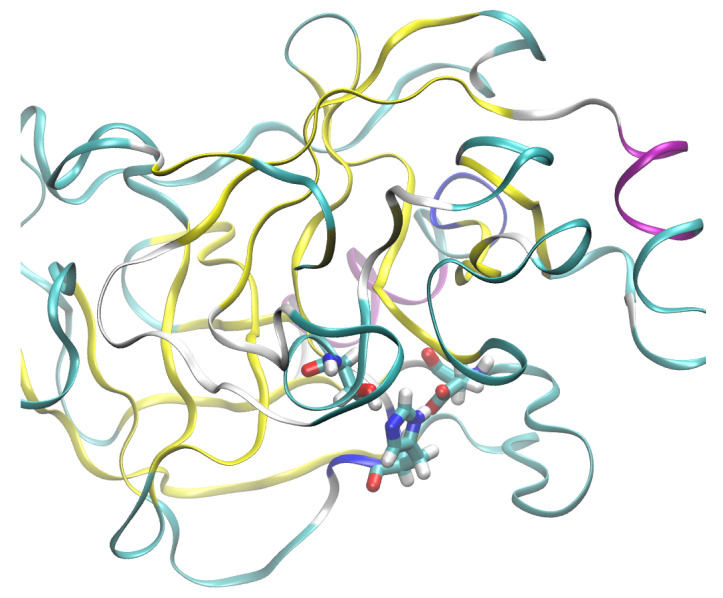
The catalytic triad of the studied cationic trypsin shown in licorice representation: Ser195–His57–Asp102. Exposure of the triad to the external environment is visible. Color coding of the secondary structure: α-helix—purple; β-strand—yellow; turn—cyan; random coil—white.

The most prevalent secondary structure motif in trypsin is the β-strand, while the larger protein, CYP51, contains more helical domains. The surface of trypsin contains mostly basic residues, while the demethylase is more diversified with respect to the residue types. The active site of trypsin encompasses the catalytic triad, Ser195–His57–Asp102 (see Figure 2). Structural modifications to this site will directly impact the ability of this enzyme to protonate a substrate. In the following study, we will monitor the gas-phase behavior of the catalytic triad. On the other hand, our interest in the sterol 14α-demethylase is caused by its versatility in processing diverse ligands containing heterocyclic rings [32]. Further, this protein belongs to the enzyme family present in all kingdoms of life [33]. Its action, when combined with carefully designed ligands, might be associated with externally controllable phenomena (e.g., of electrostatic origin or induced by light), allowing this protein to be a part of a nanochemical device.

### 2.2. Gas-Phase Optimizations: Convergence and Structural Stability Issues

The structural models constructed on the basis of the PDB deposits 6B6T and 5FSA were subjected to optimization using the following approaches: the AM1 and PM3 semiempirical Hamiltonians (for the 6B6T structure only), the GFN1-xTB and GFN2-xTB DFTB schemes, and the GFN-FF force field. The problems encountered with the optimization can be grouped into the following categories:

- The GFN1-xTB Hamiltonians were not able to converge the self-consistent field equations despite many attempts to modify the convergence treatment (in terms of the required accuracy of the SCF, Broyden damping, etc.).
- Using the AM1 Hamiltonian for trypsin, the first SCF calculation converged in 3864 cycles, which is more than the default number of allowed SCF cycles. Further optimization required 3289 steps.
- Several AM1 and PM3 runs with modified coordinates failed after several hundreds of optimization steps due to failure of the SCF (a lack of convergence), which signified that a structural collapse had occurred.
- The GFN2-xTB Hamiltonian was able to converge the initial SCF and perform further structural optimization of trypsin, but the initial convergence was possible only with a very specific SCF setup. The initial guess was set to the Goedecker type (the Gasteiger charge and superposition of the atomic densities failed to yield converged results), and the Broyden damping was set to 0.02.
- The GFN-FF force-field setup and optimization were carried out without problems; the GFN-FF turned out to be the only successful technique for the steroid demethylase.

The failure of AM1 and PM3 optimization was accompanied by very modest Root Mean Square Deviation (RMSD) values. AM1 optimization of trypsin failed with a non-H RMSD of 0.31 Å and an all-atom RMSD of 0.36 Å; thus, the possible structural collapse was local only. We pinpointed its cause to be the dissociation of the carboxyl group from Asn74 and proton capture by the neighboring Asp153. The same residues were also responsible for the failure of PM3 optimization. Thus, these amino acids, exposed on the protein’s surface, could hamper applications of trypsin in gas-phase scenarios. It is worth mentioning that proton transfer phenomena, such as that observed for the mentioned residues, are the main factors responsible for protein refolding in the gas phase [20]. Extensive computational studies of gas-phase proton stripping events have identified striking cases of inside-out refolding [22]. Even if the protein can refold back upon rehydration into its native solution fold, the gas-phase enzymatic activity—one of the objects of interest in future applications of gas-phase macromolecular systems—would be disrupted.

**Figure 3:**
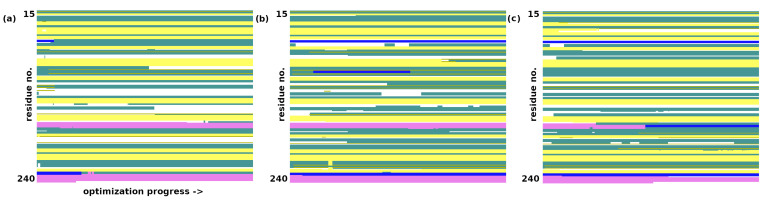
Secondary structure changes in the structural optimization of trypsin. (**a**) AM1 optimization. (**b**) GFN2-xTB optimization. (**c**) GFN-FF optimization. Color coding of the secondary structure: α-helix—purple; 3_10_-helix—blue; β-strand—yellow; turn—cyan; random coil—white.

The converged optimization of trypsin at the AM1 level proceeds with a larger final RMSD of 1.71 Å for heavy atom optimization and 1.84 Å for full minimization. The secondary structure’s elements were mostly preserved—see Figure 3. However, the structure of the catalytic triad was disrupted—even if the His57-N–H⋯O–Asp102 distance grew only to 3.14 Å, Ser195-O–H⋯N–His57 increased to 3.98 Å, effectively disrupting the hydrogen bonding network within the triad and the protein’s function on the whole.

The GFN2-xTB optimization of trypsin did not face any issues related to Asp153 and Asn74; the two residues conserved their salt bridge. No other structural collapses were found. The non-H RMSD was 1.13 Å, and the all-atom RMSD was 1.23 Å. The catalytic triad was mostly preserved, but the hydrogen bonding strength grew in the gas phase: the Ser195-O–H⋯N–His57 distance decreased from the initial 3.09 Å to 2.64 Å, while the His57-N–H⋯O–Asp102 distance grew only slightly from the initial 2.69 Å to 2.78 Å. The preservation of the overall secondary structure was also better than that for the AM1 run (see Figure 3). The importance of this result stems from the fact that the GFN2-xTB approach is quantum-chemical in its nature; thus, it takes polarization effects into account and is not dependent on the assumed topology—these two facts provide its advantage over the classical, fixed-topology force fields, although at a significantly increased computational cost. Movable proton force fields [15,16] are the middle ground—they require careful parameterization but are very fast.

The GFN-FF force-field optimization did not raise any structural issues; the problematic salt bridge between the residues Asn74 and Asp153 was conserved. It must be stressed that the nature of the force field prevents proton transfer events, but we believe that the GFN-FF result follows the GFN2-xTB description correctly. The RMSD values are larger (the non-H RMSD = 1.56 Å; the all-atom RMSD = 1.63 Å) than those in the GFN2-xTB DFTB quantum-chemical calculations, and the overall secondary structure (Figure 3) is more perturbed. Some α-helices are converted into denser 3_10_ forms, and the protein becomes more compact. The structure of the catalytic triad is conserved with a quality similar to that of the GFN2-xTB method: the Ser195-O–H⋯N–His57 distance decreased from an initial 3.09 Å to 2.94 Å, while the His57-N–H⋯O–Asp102 distance grew from an initial 2.69 Å to 2.86 Å.

**Figure 4:**
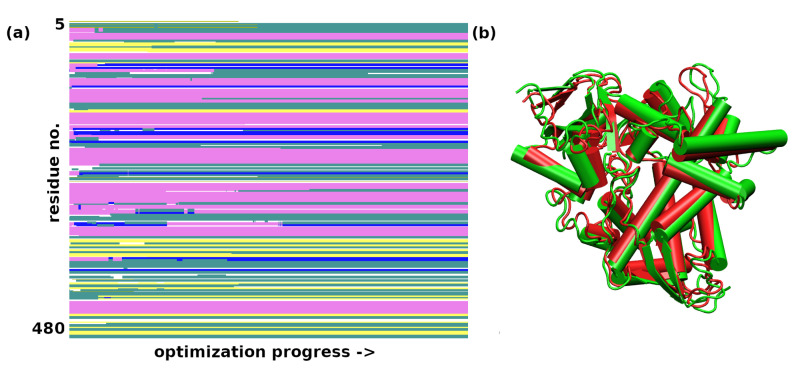
(**a**) Secondary structure changes in the structural optimization of sterol 14α-demethylase during the GFN-FF optimization. Color coding of the secondary structure: α-helix—purple; 3_10_-helix—blue; β-strand—yellow; turn—cyan, random coil—white; (**b**) A comparison of the first (green) and last (red) frame of the force-field optimization.

As mentioned earlier, the optimization using a classical force field was the only successful setup for the sterol 14α-demethylase. Figure 4 shows that the secondary structure of the protein is mostly conserved, with some instances of helical regions becoming turns. The protein becomes more compact, but the deviations in the atomic positions are not large: the non-H RMSD is 2.37 Å, and the all-atom RMSD is 2.43 Å. Reports from the literature indicate that the results of structural optimization for this enzyme, as well as for trypsin, do not have to correspond to the gas-phase conformational preference, but they could equally well result from the kinetic trapping of the solution structure within particularly large potential energy barriers. A recent computational study on ubiquitin [22] showed that the gas-phase conformational space of this 76-residue protein showed a preference for the charged residues being buried inside, exactly the opposite of the behavior in water. However, careful desolvation methods have been developed which maximize the possibility of retaining the solution-phase structure; therefore, computational studies of proteins’ behavior in the gas phase can use, as a starting point, the structures derived from solutions or crystals.

It should be emphasised that the scale of quantum-chemical, semiempirical, and DFTB calculations is large. The AM1 and PM3 calculations for trypsin employed 8399 orbitals (4686 occupied). The GFN1-xTB optimization was carried out for 9770 orbitals, and the GFN2-xTB runs were somewhat smaller, at 8178 orbitals. This makes controlling the convergence progress very tedious and slow since the energy values oscillate strongly before locking onto the SCF solution. The reason for this is that the HOMO-LUMO gap is close to zero. A recommendation on the choice of semiempirical family (the MNDO descendants—AM1 and PM3—or the DFTB GFNn-xTB approaches) cannot easily be given—both families exhibit convergence problems. However, the DFTB GFNn-xTB versions are preferred since they are newer and have been designed with dispersion corrections and other non-covalent interactions already in mind [34].

### 2.3. Molecular Dynamics with the GFN-FF Force Field

**Figure 5:**
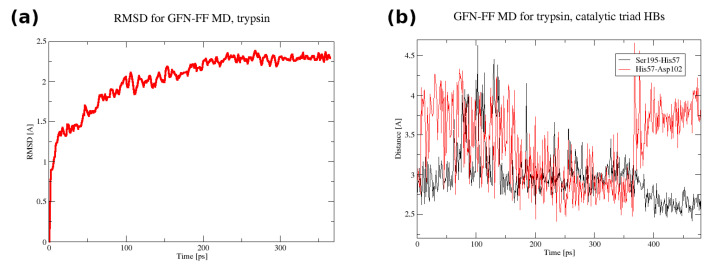
(**a**) The RMSD in the first 365 ps of the GFN-FF MD run for trypsin. (**b**) The donor–acceptor distance time evolution for the catalytic triad hydrogen bonds in the full 480 ps GFN-FF MD run for trypsin.

**Figure 6:**
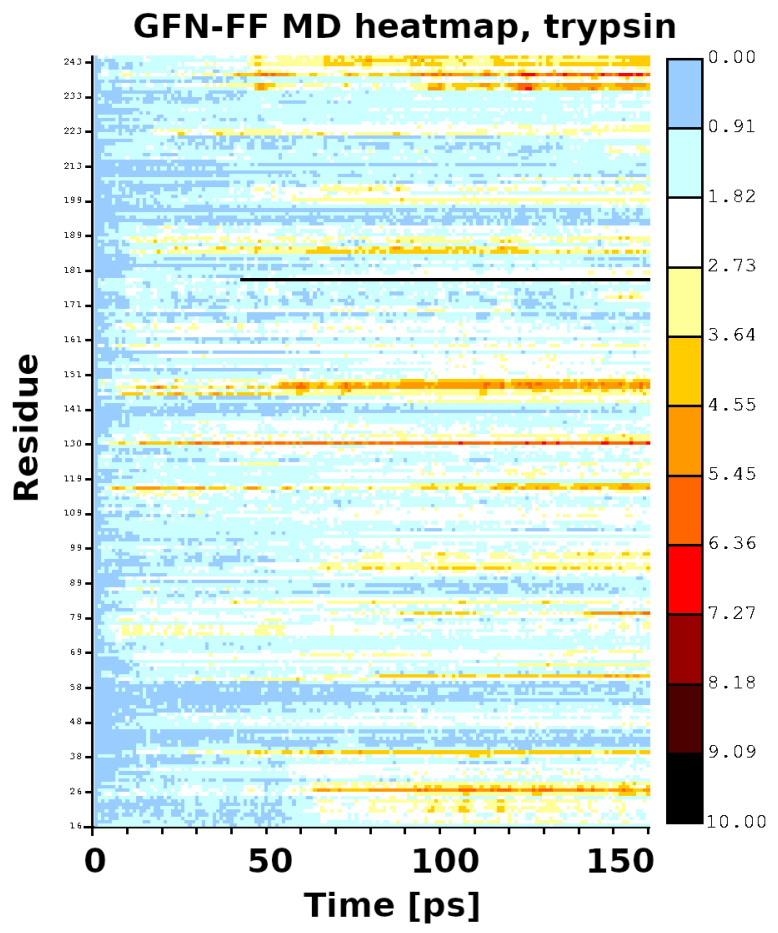
Heatmap for the first 140 ps of the GFN-FF MD run of trypsin.

The initial coordinates for the molecular dynamics (MD) runs were taken from structures optimized with the GFN2-xTB scheme for trypsin and the GFN-FF for demethylase. The use of the quantum-chemical GFN2-xTB approach allowed for an initial assessment of possible tautomerism within the protein upon the transition from the experimental condensed-phase structure to the vacuum simulation. The MD simulations were not planned to reproduce the long gas-phase trajectory of the studied biomolecules. The MD was carried out rather to pinpoint the residues immediately beginning to become “hot”, which could indicate oncoming structural failures. Indeed, at a 140 ps simulation time, the visualization of the trajectory of trypsin indicated point defects (single atoms escaping the protein), but the RMSD continued to be stable up to 360 ps. This was accompanied by structural conservation of the catalytic triad, including its hydrogen bonds (HBs)—see Figure 5. The heatmap (Figure 6) indicates that the most mobile parts of the protein already early in the simulation are close to residues 27, 39, 117, 130, 147, 179, 236, and 239. These residues are located on the protein’s surface but are not associated with any particular protein region. They are rather evenly distributed on the globular surface of the protein.

**Figure 7:**
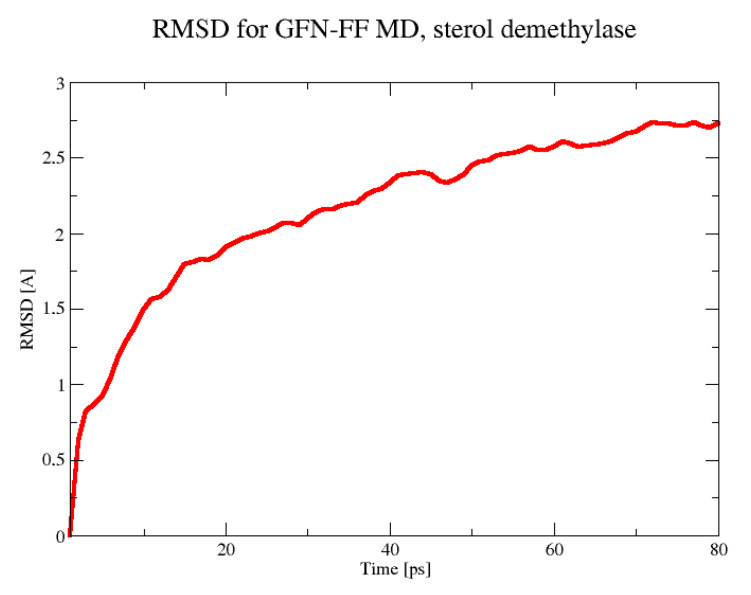
The RMSD in the 80 ps GFN-FF MD run for the sterol demethylase.

**Figure 8:**
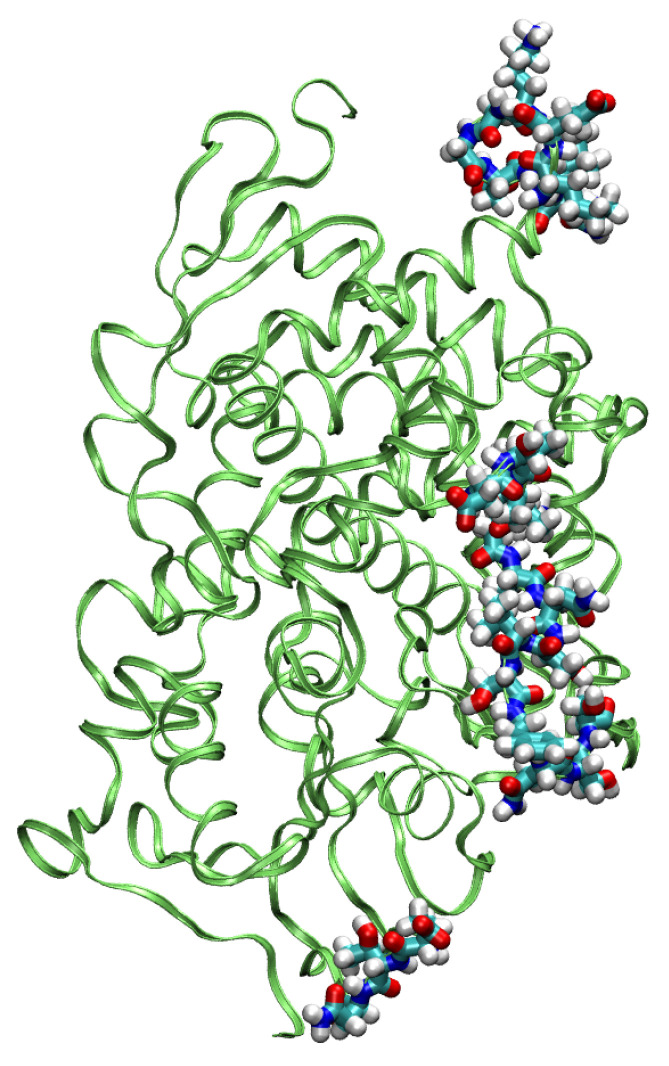
The most mobile residues in the GFN-FF MD simulation of the sterol demethylase.

The MD simulation for the sterol 14α-demethylase CYP51 was stable for a shorter period of 80 ps. Within this time, a plateau in the RMSD parameter was almost reached (Figure 7), and the most mobile parts of the protein, as evidenced by the analysis of the trajectory with the VMD program, were grouped around residues 298–305, 347–349, and 384–398. As seen in Figure 8, they formed an interface on one side of the protein. At this time scale, no disruption of any secondary structure elements was observed.

Summarizing this part of the study, we note that the use of the GFN-FF force field, which is compatible in the conceptual layer with the available quantum-chemical schemes (GFN2-xTB), makes the whole protocol more consistent. A possible combined protocol utilizing the best of both the classical and quantum approaches for further studies of proteins in the gas phase would be an initial structural study using the GFN2-xTB method and then a molecular dynamics run with the partially polarizable GFN-FF force field. This type of calculation should be viewed not as a replacement but rather as a complement to long-scale classical force-field studies. The force fields derived for solution conditions can be used for gas-phase protein modeling with the proper care and treatment of electrostatics, yielding results of a semi-quantitative nature [17]. However the literature [17] indicates the need for dedicated gas-phase MD force fields already in the near future, and their calibration with fast quantum-chemical approaches (such as GFN2-xTB) could be a valuable alternative.

## 3. Materials and Methods

Two enzymatic proteins were taken into account in this study. The corresponding structures were obtained from the PDB database [35]: a digestive enzyme, cationic trypsin 6B6T, PDB deposit [30], and a cytochrome P450 enzyme, sterol 14α-demethylase CYP51 from *Candida albicans* 5FSA, PDB deposit [31]. The coordinates of the protein chains were extracted from the PDB files, and missing atoms (including hydrogen atoms) were added with the use of tLeap, part of the AmberTools package [36]. The protonation states for cationic trypsin 6B6T were kept the same as those in the experimental model [30], while for the CYP51 5FSA structure, the standard charge states were assumed, and the histidine tautomers were determined via combined use of the structural data, the pdb4amber and tLeap, parts of the AmberTools 2021 package [36]. Any solvent molecules and other low-molecular-weight components were discarded from the structures before further processing. The resulting models contained 3221 atoms for the 6B6T structure (a single chain) and 7799 atoms for the 5FSA deposit (a single chain taken out of the dimeric structure).

The protein structures were not solvated, according to the idea of this study. Gas-phase optimizations were undertaken for both proteins using semiempirical AM1 [25] and PM3 [26] Hamiltonians. The AM1 and PM3 calculations were carried out using the EMPIRE 2014 rev. 1950 computational engine [37]. Independent optimization runs were performed using the xTB 6.4 program [34] using the density functional tight binding GFN1-xTB [27] and GFN2-xTB [28] models, as well as the GFN-FF force field [29]. Diverse setups were tested with respect to the initial guess and SCF damping for the xTB methods, as described in the Results and Discussion. Finally, molecular dynamics runs with the GFN-FF force field were performed for both proteins at a 300 K temperature, controlled via the Berendsen thermostat [38]. The initial coordinates for the MD runs were taken from the optimized structures—GFN2-xTB for trypsin and GFN-FF for demethylase. The bond lengths were not constrained (the SHAKE algorithm was not invoked), the time step was set to 2 fs, and the hydrogen atom mass was scaled by 2.0 to provide better stability in the simulation. No equilibration phase was included since we were aiming for an analysis of such structural issues, which may have been made obsolete by the equilibration itself and been overlooked in the analysis. The results of the optimizations and the MD runs were analyzed and visualized with the help of the VMD 1.9.3 program [39]. The secondary structure assignment along the MD trajectory was carried out in VMD using the STRIDE algorithm [40].

## 4. Conclusions

Our study was focused on the gas-phase properties of two enzymatic proteins, cationic trypsin and sterol 14α-demethylase. We highlighted severe challenges in obtaining a converged self-consistent field for such large systems. However, in cases where such difficulties can be overcome, the GFN2-xTB method offers good stability for subsequent optimization. The DFTB quantum-chemical approach is too demanding to allow for longer MD trajectories, and for this purpose, we propose the GFN-FF force field. The ideal combination would be GFN2-xTB optimization followed by a GFN-FF MD run, provided that the problems with the convergence of the SCF can be resolved. The recent announcement of the ONIOM’s implementation in the xTB family of tools [41] will provide additional possibilities for testing protocols similar to that proposed herein.

The results of this study have prompted our team to investigate further details of protein behavior in the gas phase using diverse techniques, not only that presented in this work. In particular, the most important issues include the fate of the hydrogen bonding networks, the progress of desolvation processes, and the possible presence of buried water molecules inside protein cavities. Molecular dynamics is an ideal tool for studying these phenomena, allowing for the direct simulation of heat impulses or covering the protein in a thin layer of water molecules and registering the fate of this layer during the MD run. The conclusions of these studies will be duly reported.

## Data Availability

The data presented in this study are available in the article itself.

## Abbreviations

The following abbreviations are used in this manuscript:

AM1	Austin Model 1 semiempirical method
CYP51	sterol 14α-demethylase
DFT	density functional theory
DFTB	density functional tight binding
ESI-MS	electron spray ionization–mass spectrometry
FF	force field
FRET	Förster resonant energy transfer
GFN-FF	a partially polarizable force field from the GFN family of methods
GFN1-xTB	extended density functional tight binding method, version 1
GFN2-xTB	extended density functional tight binding method, version 2
HB	hydrogen bond
MD	molecular dynamics
MNDO	Modifield Neglect of Diatomic Overlap—a predecessor of AM1 and PM3
PDB	Protein Data Bank
PM3	Parametric Method 3 (semiempirical approach)
RMSD	Root Mean Square Deviation
SCF	self-consistent field
